# How Do You Manage Change in Organizations? Training, Development, Innovation, and Their Relationships

**DOI:** 10.3389/fpsyg.2018.00313

**Published:** 2018-03-15

**Authors:** Riccardo Sartori, Arianna Costantini, Andrea Ceschi, Francesco Tommasi

**Affiliations:** Department of Human Sciences, University of Verona, Verona, Italy

**Keywords:** training, development, innovation, change, change management

## Abstract

The article aims to be a reflective paper on the interconnected concepts of training, development and innovation and the potential they have in dealing with change in organizations. We call *change* both the process through which something becomes different and the result of that process. *Change management* is the expression used to define the complex of activities, functions, and tools (such as training courses) through which an organization deals with the introduction of something new that is relevant for both its survival and growth. *Training* and *development* are labels used to define those educational activities implemented in organizations to empower the competences of workers, employees and managers in the lifelong learning perspective of improving their performance. Consequently, we define *competences* as those personal characteristics that allow people to be effective in the changing contexts of both workplace and everyday life. They are also necessary in *organizational innovation*, which is the process of transforming ideas or inventions into goods or services that generate value and for which customers will pay. Training, development, and innovation are three different but interconnected functions by which organizations manage change. What is the state of the art of the literature dealing with these topics? Here, is a critical review on the matter.

## Introduction

The focus of the article is organizational innovation as a way by which companies, businesses, firms and enterprises, in one word *organizations*, manage change in the multidimensional perspective of survival, competitiveness, growth, and development (Sartori et al., 2013, 2017a; Sartori and Scalco, 2014; Ceschi et al., 2017b).

In its narrowest meaning, *organizational innovation* is the process of transforming ideas or inventions into goods or services that generate value and for which customers will pay. This is the case, for example, when a new personality test is developed in order to meet the new selection and assessment demands of an organization (Cubico et al., 2010; Ceschi et al., 2014c; Sartori et al., 2014, 2016a; Charkhabi et al., 2016). More widely, organizational innovation means the application of new and useful methods in undertaking practices of business, the organization of workplace or external relationships. This specifically reminds of the concept of *open innovation*, which is a kind of innovation process based on the cooperation between people, teams, groups, and organizations (Chesbrough, 2003, 2006). In order to be successfully achieved, any kind of organizational innovation requires proper competences. This means that the mere workforce of an organization, even when highly skilled, might not be sufficient for innovation processes that really want to keep up with a world that changes so quickly. Another element that should be taken into consideration consists in delivering training activities that allow the workforce to update and empower their personal and professional characteristics, so that workers, employees, managers and, in general, members of the organization become more and more able to generate and develop ideas for innovation. Another one is the cooperation and the collaboration between people (Pedrazza et al., 2016), both working in the same organization (*closed innovation*) and belonging to different organizations (*open innovation*), which requires trust and, in general, those relational and communication skills whose existence in the workforce is not always possible to take for granted. In fact, they are usually developed by training activities. The final element we want to mention here is the ability to listen to users and customers (Sartori et al., 2017a).

All these aspects lead to the point that organizational innovation requires competences of different kind given that innovation deals with different kinds of change and different kinds of change require different competences to be dealt with, in a circular and hopefully virtuous process according to which any change can be the stimulus for innovation, and any innovation introduces changes (Sartori and Tacconi, 2017). When speaking about competences, it is not possible not to take into account those training and development activities that should be delivered in a lifelong learning perspective for both allowing people to generate new ideas and facing changes in organizations (Sartori et al., 2015). Changes require a management of them (*change management*). What does literature say about the relationships between these concepts, that is to say, training, development, change, and innovation in organizations?

## Change in Society and Organizations

*Panta rei* is the Greek phrase (πάντα 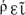) attributed to philosopher Heraclitus, which is used to express the idea that *everything flows*, everything changes. This would not be a big deal if people were not involved in change, but since the adaptation, the growth and even the survival of people depend on their ability to manage things when they have changed or are changing, the issue becomes quite relevant, even in the case, so frequent today, of changes dealing with social and relational processes. It is no coincidence that the changes related to the invention and the introduction of new technological devices (Weatherbee, 2010), most notably the Internet (Torkzadeh and Van Dyke, 2002), to the recent economic crisis (Utting et al., 2012), and to different patterns of social life (Tyler, 2002) have made scholars define our modern society as *information society* (Webster, 2002), *knowledge society* (Hargreaves, 2003), and even *liquid society* (Bauman, 2000, 2011).

As for organizations, it is well-known that their survival, growth and competitiveness in the turbulent labor market depend on their ability to manage change, both in the internal and external environment (Weick and Quinn, 1999; Sartori and Rolandi, 2013). In this framework, such a concept as “training and development” plays a recognized role for both work and personal improvement (Sartori and Tacconi, 2017), since it is regarded as a suitable response to changes (Gibbs, 2007) and a key lever for adaptation and growth (Smidt and Sursock, 2011), both individual and organizational (Roland, 2010; Western, 2010). For example, the investments that an organization puts into training and development activities contribute to creating a climate for continuous learning; and this kind of climate, as stated in Lau and Ngo (2004), stimulates a certain flow of information and ideas across employees, therefore promoting the creation of new knowledge and innovation.

Over the years, scholars have coined different expressions for “training and development” (Sartori et al., 2015), witnessed by such labels as *organizational learning* (Senge, 1990; Argyris and Schön, 1992; Fulmer and Keys, 1998), *knowledge-creating learning* (Nonaka and Takeuchi, 1995; Gherardi et al., 1998), *learning climate* (Cortini et al., 2016), *action learning* (Jones, 1990; Mumford, 1997; O’Neil, 1999), *transformative learning* (Mezirow, 1991; Hobson and Welbourne, 1998), *implicit learning* (Reber, 1993; Stadler and Frensch, 1998), *reflective learning* (Boud and Walker, 1991; Williamson, 1997), *self-directed learning* (Candy, 1991; Merriam and Caffarella, 1991), *flexible learning* (Lundin, 1999; Jakupec and Garrick, 2000) and, above all, *lifelong learning* (Moreland and Lovett, 1997; Oliver, 1999; Maehl and William, 2000).

Lifelong learning is probably the most well-known way by which the expression “training and development” is translated. It is considered the means by which people keep learning new things (Field, 2006), acquiring competences (Shandler, 2000), making meaning, gaining wisdom and expertise (Jarvis, 2009), adapting to different environments (International Labour Organization, 2000), developing while growing (Commission of the European Communities, 2007) and, in short, changing as *everything flows, panta rei*.

Lifelong learning is both a theoretical and practical concept that refers to the idea that it is both possible and necessary for human beings to keep on getting information, knowledge and competences throughout their lives for either personal or professional reasons (adaptation, improvement, growth, development, etc.). It involves such education and training activities as reading, studying, attending lessons, working, practicing at home or other places, traveling and, basically, gaining experiences of different kind (off and on-line). In fact, according to a classical definition, lifelong learning is a process through which individuals acquire information, knowledge and competences in a range of formal and informal settings, throughout life. It may occur as part of schooling, education, training, personal development (Brookfield, 1986; Grant and Stanton, 1998) or workplace-based learning (Billet, 2011).

Against this background, it becomes clear that competence is a key concept within the perspective of both lifelong learning and change management. From a theoretical point of view, over the years, the term competence has been defined in several ways (Gelman and Greeno, 1989; Elbers, 1991; Ellström, 1997; Mulder, 2007), depending on the context and the perspectives adopted (Fischer et al., 1993), whereas practically, both scholars and laymen acknowledge that it is something related to learning, training, work, and organizations (Spencer and Spencer, 1993). Competences are precisely those personal characteristics (a set of knowledge, abilities, and attitudes) that allow people to be effective in the workplace and in everyday life. Competences can be learned (McClelland, 1973; Nuthall, 1999). That is the reason why they tend to be taught through education and training activities dedicated to people working in organizations and living in our modern society (Raven and Stephenson, 2001).

The increasing importance given to the development of people in organizations has stimulated researchers to study the relationship between training activities and several performance measures (Tharenou et al., 2007; Ceschi et al., 2016, 2017c; Sartori et al., 2016b, 2017b). Empirical studies have investigated the outcomes of training on productiveness (Barrett and O’Connell, 2001), financial performance (Glaveli and Karassavidou, 2011), and motivation of employees (Castellanos and Martín, 2011). Surprisingly enough, the relation between training activities and organizational innovation has been instead widely overlooked (Nguyen et al., 2010).

## What Competences for What Organizational Innovation

Organizational innovation refers to new modalities by which work can be organized and achieved within companies, businesses, firms and enterprises to foster and promote competitive advantage. It involves organizations, groups, and people in managing work processes in such areas as customer relationships, employee performance, and retention and knowledge management. Accordingly, workplace innovation is a bundle of practices and programs involving changes in the business structure, in the human resources management, in the relationships with customers and suppliers, and/or in the work environment itself (Costantini et al., 2017a,b).

The general term of innovation is used to refer to the development and the implementation of new ideas, new devices and new processes (Sartori et al., 2013). For this, it is assimilated to creativity and originality, *creativity* being the tendency to create or identify ideas, alternatives or possibilities that can be useful in solving problems, communicating and entertaining (Sartori and Scalco, 2014), while *originality* is considered the quality of being new and different in a good and appealing way (Sartori et al., 2017a).

As stated in Sartori and Scalco (2014, p. 63), “three concepts seem to be particularly linked together: training, development, and innovation (Ceschi et al., 2014a).” For instance, innovation, creativity and originality require people to be able to generate or recognize ideas and implement them in products, services or behaviors that are not only new but also useful. In fact, as Wallin and von Krogh (2010) state, we can call innovative only those processes that cover the creation of relevant knowledge for the development and the introduction of something new and useful in organizations. Ideas for innovation, creativity and originality come from people. Therefore, people should have specific competences to generate innovative ideas. What are these competences?

While literature is always emphasizing the central role of people in innovation processes, research has not yet extensively explored the so-called *human side of innovation* (Sartori et al., 2017a). Several consulting books describe the skills and competences needed by the members of the so-called *innovation teams* in order to generate and implement new ideas, but their descriptions are derived from authors’ personal and professional experience and are not equally supported by research. Sloane (2011), for example, proposes that the competences needed in innovation processes can be categorized into two types: *hard skills* and *soft skills*. Hard skills are those competences applied to such tasks as designing activities, assessing artifacts, or managing projects. Soft skills, on the other hand, are obtained by a combination of personality traits, attitudes, and relational competences that can be largely applied across different innovation tasks and activities.

Leafing through literature, it is not always evident what kind of competences are really necessary in order to make an innovation team effective. The competences reported may not be exclusively related to the field of innovation. Apart from this, it might also be a question of skill level on which the competences are mastered. Competences and skills which are normally considered specifically related to innovation teams could be also found in other kinds of teams. Research does not show in what way the required competences are different or should be different between innovation contexts and other settings. For example, *being able to combine different points of view* is surely crucial in decision-making teams and problem-solving situations as well, but the required mastery level of this competence in the case of innovation teams may be different in both quantity and quality.

Some of the characteristics that authors suggest that people working in innovation teams should have are the following (see also Sartori et al., 2013, p. 12):

1. An *entrepreneurial mindset* (Cubico et al., 2010; Lindegaard and Kawasaki, 2010; Sloane, 2011), so that people involved in innovation teams take responsibility for and are proactive toward what they are supposed to do;
2. Solid *communication skills*, which basically means being able to combine listening and speaking skills (Shockley-Zalabak, 2008; Ceschi et al., 2014a), so that people involved in innovation teams can share and compare ideas;
3. *Ability to understand* technical requirements which are not simple in their nature and reduce them into easier elements so that the different members involved in the innovation team can better manage them (Kanter, 2006; Sloane, 2011);
4. Skills for building and maintaining *relationships*, in order to stimulate cooperation among people even in the presence of different personal characteristics (Kanter, 2006; Lindegaard and Kawasaki, 2010; Sloane, 2011);
5. *Curiosity*, as spontaneous desire to learn things of different kind and to integrate them together in order to meet or sustain the strategic targets of innovation (Lindegaard and Kawasaki, 2010);
6. *Holistic point of view*: the ability to interpret the organizational culture which has the possibility to influence the fact the innovation actually moves forward (Ritter and Gemünden, 2003).

From a psychological point of view, innovating in teams means sharing risks and rewards with others and this implies trusting the other members of the team (Shamah and Elsawaby, 2014; Salampasis et al., 2015) and collaborating with them (Sawyer, 2008; Ceschi et al., 2017d). This is not always an easy thing to achieve, as trust and collaboration are among those psychological and relational characteristics that need proper training to be developed (Sartori and Ceschi, 2013), *trust* being the belief that someone or something is reliable, good, honest, effective, etc., while *collaboration* means working with others to do a task and to achieve shared goals.

Trust, especially in the form of inter-organizational trust, or the trust between two organizations, is necessary in order to let external ideas and tools flow in from the outside and, more difficult from a psychological point of view, let internal knowledge flow to the outside. In this sense, trust is a core element of *open innovation*, which is the use of deliberate inflows and outflows of information to speed up internal innovation (Chesbrough, 2003, 2006).

Collaboration is necessary to let people openly communicate, share and cooperate and therefore benefit from different points of view, which increases the probabilities to see things differently and therefore to discover, find out and/or invent something new just through communication, sharing and cooperation. This concept is expressed in the book by Keith Sawyer entitled *Group Genius – The Creative Power of Collaboration* (Sawyer, 2008), where the author emphasizes the concept that *innovation is driven by collaboration*.

On the other hand, Lindegaard and Kawasaki (2010) claim that it is important to involve persons who can be called *innovation leaders* or *intrepreneurs* (it is just *intrepreneurs*, not *entrepreneurs*), that is to say, people able to both focus on such a strategic task as creating the internal conditions which are necessary to develop organizational innovation capabilities and drive innovation projects in spite of the challenges they have to deal with, such as uncertainty, resource availability, and differences in aims.

A wide-ranging study on the competences required to people involved in innovation teams has been carried out by du Chatenier et al. (2010). For the study, publications on inter-organizational learning, innovation and change management, business associations and networks in organizational management and human resources surveys was consulted. The competence profile derived from this literature has also been sustained by an empirical investigation. In such investigation methods as interviews and focus groups, participants were asked to express themselves on the *critical incidents* or *challenging situations* they faced in innovation settings and to describe how they managed all of this.

The challenges and competences reported were different among the respondents. The interviews gathered a large range of responses with apparently conflicting aspects related to competence. This could be the result of the fact that respondents took part in different innovation teams, with differences in partnerships, cooperation tools, and objectives. In addition, the diversity of responses may be due to the specific background and context of the interviewees. The main outcome of the investigation by du Chatenier et al. (2010) is the list of characteristics reported in **Table 1** (see also Sartori et al., 2013), which also shows the links between competences (skills), situational conditions and team (group) performance. How to develop these competences?

**Table 1:** Competences for innovation.

**Competences of extra importance in certain contexts**	
Project management	**Involve**: Identifies human, material and experiential resources for accomplishing various kinds of learning objectives. Identifies situations for participative group problem solving, using the proper degree of participation, and recognizes obstacles and corrective actions. Knows who to inform and when. **Influence**: Appropriately adapts, calibrates ones behavior to each situation in order to elicit particular responses from others. Uses influencing skills (as opposed to instructing): position, coalition, stimulation. Knows how to play the political game. **Create learning climate**: Shares success, allows people to make mistakes. Is honest: possesses high levels of integrity, authenticity, sincerity, and genuineness. Can be counted on to represent situations fairly. Develops, maintains, and uses effective networks. Is approachable, develops friendships easily and strong beneficial alliances and coalitions. Develops a team spirit. Deals with unexpected situations, is flexible with plans, deadlines, improvises. Is not too systematic, rigid. Deals with a flexible team composition.

**Both**	**Prevail**: Has an overall picture of the project and influencing factor. Understands and manages complexity. Supports many things on his/her mind at the same time. Has self confidence. Is competent: able to perform the tasks required by his or her position.

**Complex alliances**	**Take on**: Is aware of, and regulates, own thinking and feeling. Manages tensions created by multiple accountabilities, tasks and roles. Has perseverance, keeps on thinking positively, having end-goal in mind. Is reliable: ensures that the others can depend upon him/her to come through for them, acts consistently, follows through. Is pro-active. Comes up with ideas him/herself and takes initiatives. **Communicate clearly**: Creates a vision. Appreciates the learning domain and has the motivation to learn, has a sense of urgency. Is open: shares information freely with others, even when (s)he is not sure. Communicates clearly and understandably. Recognizes open and supportive communication methods.

**Competences related to team performance**	
**Positively**	**Monitor**: Coordinates and synchronizes activities, information, and tasks between team members. Designs a plan of strategies. Carries out the plan systematically and sequentially. Feels responsible for the team and acts as such. Monitors, evaluates, and provides feedback on overall team and individual performance. Accepts feedback about his/her performance non-defensively. Collects evidence of accomplishments. Asks many critical questions. Trusts the other party.

**Negatively**	**Compete**: Is critical but constructive. Is aware that (s)he represents an organization; refuses to accept less.

**Positively or negatively**	**Handle conflicts**: Openness: treats differences as important opportunities. Respects, values and appreciates people and their ideas. Possesses basic knowledge and perceptions of various technical/professional areas and business languages. Has experience working in partnerships. Is assertive, extroverted. Communicates own perceptions and feelings (in a diplomatic way). Is straightforward. **Analyze**: Wants to learn from others. Understands social situations as well as interpersonal interactions (Scalco et al., 2017). Is sensitive to the roles and responsibilities of all partners, aware of their collaborative motivations and expresses understanding and empathy. Has good reflective skills and applies techniques of lateral thinking or divergent thinking.

**Other relevant competences**	
**Relevant for all open innovation professionals**	**Decide mindfully**: Knows what his/her qualities are, does not take the position of the underdog. Possesses basic knowledge and perceptions. Establishes specific, challenging, accepted team goals. Diagnoses, formulates learning objectives in performance outcomes (but not too quickly). Is benevolent: has the best interests of others at heart. **Explore**: Combines high advocacy (egocentrism) with high inquiry. Recognizes types and sources of conflict, encourages desirable conflict but discourages undesirable conflict. Picks up signals, sees opportunities, has intuition for innovation. Balances short- and long-term goals. Identifies problems. Discerns sub from main issues. **Combine**: Employs integrative (win–win) negotiation strategies rather than distributive (win–lose) strategies. Brokers solutions or outcomes. Thinks in ways that differ from established lines of thought. Agrees to disagree (lose–lose strategy). Considers common goals mostly important. Adapts without violating own ideas.

*This table is copied from Sartori et al. (2013).*

## Training and Development for Organizational Innovation

According to the Oslo Manual published by OECD^1^ (*Organization for Economic Cooperation and Development*) OECD and Eurostat (2005), “innovation is the implementation of a new or significantly improved product (good or service), or process, a new marketing method, or a new organizational method in business practices, workplace organization or external relations” (see also Sartori et al., 2013, p. 2).

The OECD (2011) takes in consideration four kinds of innovation (Sartori et al., 2013, p. 2):

1. *Product innovation*: introduction of goods or services that are new or improved in a significant way as for their features or potential uses;
2. *Process innovation*: introduction of methods which are new or improved in the fields of production or delivery;
3. *Marketing innovation*: application of new methods of marketing with changes in product design or packaging, product placement, product promotion or pricing;
4. *Organizational innovation*: application of new methods in the business practices, the organization of the workplace or external relationships.

Van der Meer (2007) synthetizes that “innovation is the whole set of activities leading to the introduction of something new resulting in strengthening the defendable competitive advantage of an organization” (Sartori et al., 2013, p. 3), while the Oslo Manual specifies that innovation can be (Sartori et al., 2013, p. 3):

1. *new to the organization*: it may have already been implemented by other organizations, but it is new to one specific organization;
2. *new to the market*: an organization is the first to introduce it in the market;
3. *new to the world*: an organization is the first to introduce it for all markets and organizations.

From these lines, it is possible to derive two different principles for innovation, which are to be considered in training and development activities. The first one is that innovation is often the result of the ability to make use of existing knowledge and information to give birth to different combinations and reconfigurations (Cantner et al., 2008). The second one, already stressed in this paper, is that innovation encompasses the cooperation of people and groups with different knowledge, experience and expertise (human and psychological capital).

Kelley (2010) emphasizes that innovation is not accomplished by a single skilled worker, but can only be achieved in cooperation (Sartori and Scalco, 2014, p. 68): “While many people give Thomas Edison, Alexander Graham Bell, and the modern-day equivalent, Dean Kamen, credit for being lone inventors, the fact is that the lone inventor myth is just that – a myth. All these gentlemen had labs full of people who shared their passion for creative pursuits.” In fact, innovation appears to be the outcome of three social activities described as follows (Kelley, 2010; Sloane, 2011; Sartori et al., 2013, p. 3):

(1) *Social inputs* – In the first place, organizations try to recognize key insights for innovation. Through such social research techniques as focus groups and ethnographic inquiries or links to other organizations and disciplines, they try to collect insights and be inspired.
(2) *Social evolution* – Organizations make use of innovation teams and groups, not sole inventors, to convert key insights and find new solutions.
(3) *Social execution* – It comprises such social outputs as trials, beta programs and trade shows. It is crucial for customers to be trained so that they can recognize their necessities for innovation. Henry Ford summed up this problem with his famous quote “If I had asked people what they wanted, they would have said: faster horses.”

According to Ferrary (2011), innovation would pass through a life cycle defined as an interactive process that begins with *exploration* and finishes with *exploitation* (Sartori et al., 2013, p. 3). Exploration is considered to be the phase leading to knowledge generation, while exploitation happens when the knowledge that brings up innovation is finally industrialized and commercialized. Both the steps, exploration and exploitation, are dependent on human and (positive) psychological capital (Sartori et al., 2013; Sartori and Scalco, 2014). According to the OECD (2011, p. 18), human capital is defined as “the knowledge, skills, competences, and attributes embodied in individuals that facilitate the creation of personal, social, and economic well-being.” On the other hand, “positive psychological capital is defined as the positive and developmental state of an individual as characterized by high self-efficacy (Bandura, 1997), optimism, hope and resiliency” (Luthans and Youssef, 2004; Luthans et al., 2004; Salanova et al., 2012; Sartori et al., 2013).

It is important to get an idea of what human and psychological factors foster or hinder the collaborative knowledge creation, in order to design training courses able to develop them. Scholars have repeatedly claimed that training practices improve innovation by endorsing learning climate (Gómez et al., 2004; Shipton et al., 2005; Cortini et al., 2016) and exploratory learning (Shipton et al., 2006; Beugelsdijk, 2008), while, not unexpectedly, research shows that the way partners cope with the collective learning processes, communicate and cooperate plays an essential role in the success of strategic collaborations for new products and services (Larsson et al., 1998; Ceschi et al., 2014b, 2017e, 2018; Manuti et al., 2017; Scalco et al., 2018). Research also shows that people need to be trained on these issues since you cannot assume that people know how to do this spontaneously (Sartori and Scalco, 2014). There is an abundant research literature on the question of what makes teams and work groups effective in the case of innovation.

A meta-analysis by Hülsheger et al. (2009) on the topic of team-level antecedents of creativity and innovation in the workplace examined 15 team-level variables and their link to creativity and innovation. An exploration of the innovation literature dealing with training and development resulted in a final sample of 104 studies. Results revealed that such team process variables as *support for innovation, vision, task orientation*, and *external communication* displayed the strongest correlations (*r*) with creativity and innovation (*r*-values between 0.40 and 0.50), while input variables (i.e., team composition and structure) showed weaker effect sizes. Therefore, the authors conclude, it is worth training and developing people on those team-level aspects.

Again, an article published by Ceschi et al. (2014a) presents the results of a longitudinal study conducted during 4 months with 183 Italian participants, divided into 50 teams of three (*n* = 24), four (*n* = 19), and five (*n* = 7) members. Participants were involved in a business game in which the aim was not only to earn virtual money, but also to learn long-term strategies to develop profitable investments without losing sight of economic factors. The study investigated the *communication and innovation* (CI) *dimension* drawn from the Italian version of the team climate inventory (TCI) by Ragazzoni et al. (2002). An *r-*value of 0.301 (*p* = 0.048) between team performance and CI was found, while input variables (i.e., team composition and structure) showed no effect at all, which is consistent with the results found with the meta-analysis by Hülsheger et al. (2009).

Finally, a study by Loewen and Loo (2004) clearly shows that the concept of internal communication measured by the TCI is in relation to group climate, organizational learning and group performance in terms of innovation outputs.

All these studies presume that the learning processes are the underlying mechanisms that account for the effects of training on innovative performance (Laursen and Foss, 2003; Chen and Huang, 2009), strongly underline the importance of human resources training in developing the characteristics that literature has shown to be in relation to creativity and innovation processes (Sartori and Scalco, 2014), but they do not arrive to a clear training and development model to be delivered to people involved in such processes (Sartori et al., 2017a). Instead, they identify in the psychosocial training the means by which it is possible to obtain the development of the characteristics needed by people dealing with innovation, without specifying how all this should work.

**FIGURE 1:**
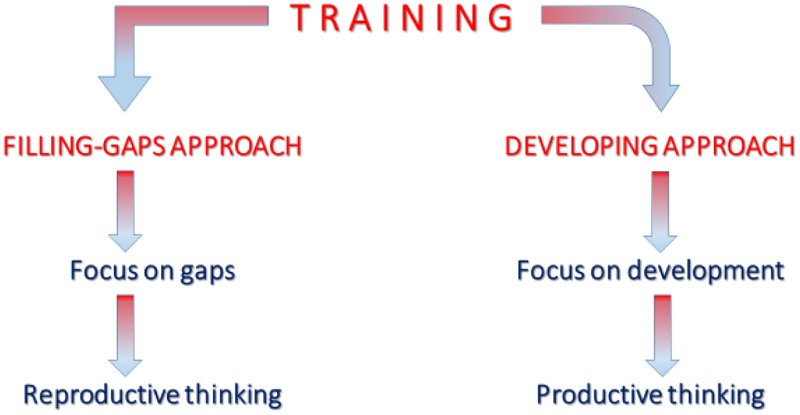
The Filling-gaps approach and the Developing approach to training. The latter seems to be a more effective approach to training for innovation than the former.

A framework that can be used to have an idea of the kind of training and development, which is possible to deliver in the case of innovation teams, is the one shown in **Figure 1**. In it, two different approaches to training are shown: a *filling-gaps approach* and a *developing approach*. The first one can be linked to the targets of combinations and reconfigurations expressed earlier (Cantner et al., 2008), while the latter seems to be a more effective approach to training for innovation than the former, since the developing approach is connected to a more creative productive thinking (which combines knowledge with creative/critical thinking) than the filling-gaps approach, which is connected to a less original reproductive thinking (which is simply a way to refine what is already known). In addition, the filling-gaps approach can be used for training practices dedicated to just one person (through off-line activities such as a handbook or on-line experiences such as a tutorial), while the developing approach can be achieved only in group, which makes this kind of training practice more suitable for sharing ideas and developing the ability to cooperate.

## Conclusion and Further Considerations

Although most of the consulting books and dissemination publications emphasize the importance of the so-called *human factor* in innovation processes that really want to keep up with a world that changes so quickly, little research on the implications of training people has been carried out in order to understand how to properly develop them in the perspective of organizational innovation. In fact and so far, the relationship between training practices and organizational innovation has been widely overlooked (Nguyen et al., 2010) and the effect of “training and development” on organizational innovation has yet to be studied (Tharenou et al., 2007). In addition, theoretical elaboration and empirical evidence remain lacking (Sung and Choi, 2014). This is rather surprising especially if we focus on the importance that the organization’s innovative capability has in achieving competitive advantage and sustainable growth (Lau and Ngo, 2004).

The study by Sung and Choi (2014) is one of the few ones examining the effects of training and development activities on organizational innovation. The authors specifically suggest that the training and development investments of an organization affect its innovative performance by promoting several learning practices (as the ones listed at the beginning of this paper). They empirically tested their hypothesis by using time-lagged, multi-source data collected from 260 Korean companies that represent various industries. Their analysis shows that corporate expenditure for internal training predicts interpersonal and organizational learning practices, which, in turn, increase innovative performance. The data also reveal that the positive relationship between interpersonal and organizational learning practices and innovative performance is stronger within organizations that have stronger innovative climates. The study provides a plausible explanation for a mechanism through which the investments of an organization in employees enhance its innovative performance but does not provide the way by which people in organizations should be trained in order to give birth to innovation processes.

What is sure, though, is that, in order to guarantee a successful implementation of organizational innovation practices, it is essential to comprehend what are the elements, the factors and the dimensions that allow two or more parties to build up a mutual working relationship, which is important in closed innovation and necessary in open innovation. Following Whelan et al. (2011), when the aim is to let outside ideas enter the organization and arrive to the people best equipped to take advantage of them, *idea scouts* and *idea connectors* should be nominated inside the organization. Whelan et al. (2011) define *idea scouts* as “the antennae” of the Research & Development (R&D) Departments. Their main task is to assemble the signals on emerging scientific and technological developments spread out all over the world. On the other hand, *idea connectors* are the people who, inside the organization, can count on an extensive network of people. They should have the know-how required to allocate the technological information collected. *Connectors* can be considered the center of the organization’s social network. Their main expertise lies in knowing *who is doing what*. They should have the ability to decipher external information into a form understandable and relevant for internal colleagues. Finally, they should also be able to convince other network members to take the actions required to give birth to innovation.

The model we have just presented is supposed to foster the implementation of both closed and open innovation strategies. Leaving apart the fact that some organizations appoint strategic job roles to single employees, as it is the case of idea scouts and connectors, other companies tend to implement innovation processes through (*open*) *innovation teams*, which are composed of different professionals coming from different organizations with the common objective to integrate knowledge in order to implement new products or services (Hafkesbrink and Schroll, 2010). The studies carried out so far on the matter demonstrate that the cooperation with external partners is particularly challenging and difficulties should not be underestimated (du Chatenier et al., 2010; Ceschi et al., 2014c). For example, studies on teams stress the idea that “while working in teams can potentially create synergies so that the team produces an output which is better than could have achieved by any individual member working alone, teams can also produce outputs which are worse than could have been produced by the most competent team member” (Newell and Swan, 2000, p. 1291).

Some of the possible problems related to teamwork are: *conformity* and *obedience* (Asch, 1956; Milgram, 1965), which make people give up expressing their own ideas; *groupthink* (Janis, 1972), which leads people to quickly converge toward one idea without appropriately exploring other possibilities; and *group polarization* (Isenberg, 1986), which results in ingroup–outgroup dynamics; but open innovation teams are confronted with further problems, such as *finding external partners* (Omta and van Rossum, 1999; Salter et al., 2014). The selection of external cooperators requires a careful evaluation of partners’ characteristics and a punctual analysis of potentials and risks referring to the collaboration. Once the cooperation is established, (open) innovation teams find themselves facing such problems as *overcoming cognitive distances* (Cohen and Levinthal, 1990), the risk of *uncontrolled disclosure* or *leakage of information* due to the difficulty of balancing individual and alliance interests (Hamel, 1991), *lack of trust* (Doz and Hamel, 1998) and *unequal power distribution* (Falk and Falk, 1981).

These are some of the most important reasons why people involved in innovation teams should be properly trained in order to be able to cooperate and generate innovation in organizations through teamwork and collaboration. In fact, and we want to stress the idea once again, organizational innovation requires competences of different kind given that innovation deals with different kinds of change and different kinds of change require different competences to be dealt with, in a circular and hopefully virtuous process according to which any change can be the stimulus for innovation, and any innovation introduces changes (Sartori and Tacconi, 2017). Accordingly, organizational innovation needs ideas, ideas are generated by people and these ideas are influenced by the so-called human and psychological capital supplied to humans: knowledge, skills, competences (OECD, 2011), self-efficacy (Bandura, 1997), optimism, hope, and resiliency (Luthans and Youssef, 2004; Luthans et al., 2004; Ceschi et al., 2017a), all characteristics whose existence cannot be taken for granted in the workforce and that, consequently, should be developed by proper training activities in order to let people develop the competences needed to work with others in the perspective of generating ideas and transforming them into innovative (new and useful) ideas.

The way to do this is still open to investigations and reflections.

## Author Contributions

All authors listed have made a substantial, direct and intellectual contribution to the work, and approved it for publication. RS is responsible for the title, the abstract and the general idea of the paper. ArC and AnC have contributed with their previous papers and new ideas in all the sections of the paper. FT is in particular responsible for the introduction and the conclusions.

## Conflict of Interest Statement

The authors declare that the research was conducted in the absence of any commercial or financial relationships that could be construed as a potential conflict of interest.

## Footnotes

1 It is composed of the following Countries: Australia, Austria, Belgium, Canada, Chile, Czechia, Denmark, Estonia, Finland, France, Germany, Greece, Hungary, Iceland, Ireland, Israel, Italy, Japan, Korea, Luxembourg, Mexico, Netherlands, New Zealand, Norway, Poland, Portugal, Slovakia, Spain, Sweden, Switzerland, Turkey, United Kingdom, United States.
